# Supplementary material for: Machine learning-based in-hospital mortality prediction of HIV/AIDS patients with Talaromyces marneffei infection in Guangxi, China
Source: PLoS Negl Trop Dis. 2022 May 4;16(5):e0010388. doi: 10.1371/journal.pntd.0010388 (PMC9067679; doi:10.1371/journal.pntd.0010388)
Supplement: S1 Table — ART, antiretroviral therapy, a Kolmogorov-Smirnov, b Chi-square test, c t-test. (DOCX) [file pntd.0010388.s001.docx]

S1 Table. General characteristics of 1927 HIV/AIDS patients with T.marneffei infection at the Fourth People's Hospital of Nanning, Guangxi

|  |  |  | Outcome | | |  |  |  |
| --- | --- | --- | --- | --- | --- | --- | --- | --- |
| Characters | Total (n=1927) |  | Survival (n=1671) |  | Death (n=256) |  | Statistic | *p-*value |
| Age (years) | 43(35,54) |  | 43(36,54) |  | 42(34,55) |  | -1.020^a^ | 0.308 |
| Time of in-hosptital (days) | 20(11,28) |  | 21(13,29) |  | 8(3,17) |  | -13.078^a^ | <0.001 |
| BMI (kg/m^2^) | 18.76±2.84 |  | 18.81±2.79 |  | 18.39±3.19 |  | 1.496^b^ | 0.137 |
| Sex |  |  |  |  |  |  | 0.956^c^ | 0.328 |
| Male | 1585 |  | 1380(87.1%) |  | 205(12.9%) |  |  |  |
| Female | 342 |  | 291(85.1%) |  | 51(14.9%) |  |  |  |
| Nationality |  |  |  |  |  |  | 6.772^c^ | 0.034 |
| Han | 1147 |  | 977(85.2%) |  | 170(14.8%) |  |  |  |
| Zhuang | 736 |  | 657(89.3%) |  | 79(10.7%) |  |  |  |
| Other | 44 |  | 37(84.1%) |  | 7(15.9%) |  |  |  |
| Occupation |  |  |  |  |  |  | 15.300^c^ | <0.001 |
| Farmer | 1061 |  | 949(89.4%) |  | 112(10.6%) |  |  |  |
| Unemployed | 361 |  | 300(83.1%) |  | 61(16.9%) |  |  |  |
| Other | 505 |  | 422(83.6%) |  | 83(16.4%) |  |  |  |
| Marital status |  |  |  |  |  |  | 7.313^c^ | 0.026 |
| Married | 1146 |  | 1013(88.4%) |  | 133(11.6%) |  |  |  |
| Single | 727 |  | 611(84.0%) |  | 116(16.0%) |  |  |  |
| Other | 54 |  | 47(87.0%) |  | 7(13.0%) |  |  |  |
| ART before admission |  |  |  |  |  |  | 14.276^a^ | <0.001 |
| No | 1374 |  | 1166(84.9%) |  | 208(15.1%) |  |  |  |
| Yes | 553 |  | 505(91.3%) |  | 48(8.7%) |  |  |  |

ART, antiretroviral therapy, ^a^ Kolmogorov-Smirnov, ^b^ Chi-square test, ^c^ t-test
