# Supplementary material for: Machine learning-based in-hospital mortality prediction of HIV/AIDS patients with Talaromyces marneffei infection in Guangxi, China
Source: PLoS Negl Trop Dis. 2022 May 4;16(5):e0010388. doi: 10.1371/journal.pntd.0010388 (PMC9067679; doi:10.1371/journal.pntd.0010388)
Supplement: S2 Table — IRIS, immune reconstitution inflammatory syndrome. (DOCX) [file pntd.0010388.s002.docx]

S2 Table. Effects of different clinical complications/coinfections on the mortality of 1956 HIV/AIDS patients with T.marneffei infection at admission

| Complications/coinfections | Total no. of patients | Death. N | Person-months | Death/100Person-months (95%CI) | *p*-value |
| --- | --- | --- | --- | --- | --- |
| Fever |  |  |  |  | 0.407 |
| No | 940 | 132(51.6%) | 676.4 | 19.5(16.3-22.8) |  |
| Yes | 643 | 76(29.7%) | 468.7 | 16.2(12.7-19.8) |  |
| Missing | 344 | 48(18.8%) | 200.3 | 24.0(13.9-34.0) |  |
| Pneumonia |  |  |  |  | <0.001 |
| No | 623 | 44 (7.1%) | 422.4 | 10.42 (7.42-13.42) |  |
| Yes | 1304 | 212 (16.3%) | 922.9 | 22.97(19.96-25.99） |  |
| Tuberculosis |  |  |  |  | <0.001 |
| No | 1190 | 178(69.5%) | 760.3 | 23.4(20.1-26.8) |  |
| Yes | 737 | 78(30.5%) | 585.1 | 13.3(10.4-16.2) |  |
| Lymphatic tuberculosis |  |  |  |  | 0.350 |
| No | 1910 | 255(99.6%) | 1330.9 | 19.2(16.9-21.5) |  |
| Yes | 17 | 1(0.4%) | 14.5 | 6.9(-6.3-20.1) |  |
| Pneumocystis |  |  |  |  | 0.011 |
| No | 1674 | 209(81.6%) | 1171.0 | 17.8(15.5-20.2) |  |
| Yes | 253 | 47(18.4%) | 174.4 | 26.9(19.4-34.5) |  |
| Oral fungal |  |  |  |  | 0.658 |
| No | 1012 | 131(51.2%) | 704.1 | 18.6(15.5-21.7) |  |
| Yes | 915 | 125(48.8%) | 641.3 | 19.5(16.2-22.8) |  |
| Cryptococcus |  |  |  |  | 0.276 |
| No | 1912 | 255(99.6%) | 1328.5 | 19.2(16.9-21.5) |  |
| Yes | 15 | 1(0.4%) | 16.9 | 5.9(-5.4-17.3) |  |
| Herpesvirus |  |  |  |  | 0.136 |
| No | 1897 | 255(99.6%) | 1322.3 | 19.3(17.0-21.6) |  |
| Yes | 30 | 1(0.4%) | 23.0 | 4.3(-4.0-12.6) |  |
| Syphilis |  |  |  |  | 0.792 |
| No | 1872 | 249(97.3%) | 1303.2 | 19.1(16.8-21.4) |  |
| Yes | 55 | 7(2.7%) | 42.1 | 16.6(4.6-28.6) |  |
| Cytomegalovirus |  |  |  |  | 0.057 |
| No | 1586 | 220(85.9%) | 1089.2 | 20.2(17.6-22.8) |  |
| Yes | 341 | 36(14.1%) | 256.2 | 14.1(9.6-18.5) |  |
| Electrolyte disturbances |  |  |  |  | <0.001 |
| No | 1549 | 174(68.0%) | 1089.0 | 16.0(13.7-18.3) |  |
| Yes | 378 | 82(32.0%) | 256.4 | 32.0(25.2-38.7) |  |
| Hypoproteinemia |  |  |  |  | <0.001 |
| No | 1587 | 178(69.5%) | 1120.2 | 15.9(13.6-18.2) |  |
| Yes | 340 | 78(30.5%) | 225.1 | 34.6(27.1-42.1) |  |
| IRIS |  |  |  |  | 0.219 |
| No | 1880 | 252(98.4%) | 1306.4 | 19.3(17.0-21.6) |  |
| Yes | 47 | 4(1.6%) | 39.0 | 10.3(0.5-20.1) |  |
| Bronchitis |  |  |  |  | 0.578 |
| No | 1914 | 255(99.6%) | 1336.3 | 19.1(16.8-21.4) |  |
| Yes | 13 | 1(0.4%) | 9.1 | 11.0(-10.0-32.1) |  |
| Hepatitis (B or C) |  |  |  |  | 0.187 |
| No | 1636 | 223(87.1%) | 1129.3 | 19.7(17.2-22.3) |  |
| Yes | 291 | 33(12.9%) | 216.1 | 15.3(10.2-20.4) |  |
| Enteritis |  |  |  |  | 0.075 |
| No | 1827 | 236(92.2%) | 1273.7 | 18.5(16.2-20.8) |  |
| Yes | 100 | 20(7.8%) | 71.6 | 27.9(16.0-39.9) |  |
| Dermatitis |  |  |  |  | 0.007 |
| No | 1825 | 250(97.7%) | 1250.2 | 20.0(17.6-22.4) |  |
| Yes | 102 | 6(2.3%) | 95.2 | 6.3(1.4-11.2) |  |
| Hypertension |  |  |  |  | 0.447 |
| No | 1892 | 253(98.8%) | 1321.5 | 19.1(16.8-21.4) |  |
| Yes | 35 | 3(1.2%) | 23.8 | 12.6(-1.3-26.5) |  |
| Diabetes |  |  |  |  | 0.180 |
| No | 1893 | 254(99.2%) | 1318.0 | 19.3(17.0-21.6) |  |
| Yes | 34 | 2(0.8%) | 27.4 | 7.3(-2.6-17.2) |  |
| Respiratory failure |  |  |  |  | <0.001 |
| No | 1847 | 201(78.5%) | 1311.2 | 15.3(13.3-17.4) |  |
| Yes | 80 | 55(21.5%) | 34.2 | 160.8(119.4-202.3) |  |
| Septic shock |  |  |  |  | <0.001 |
| No | 1825 | 170(66.4%) | 1313.0 | 12.9(11.0-14.8) |  |
| Yes | 102 | 86(33.6%) | 32.3 | 266.0(211.2-320.8) |  |
| Tumors |  |  |  |  | 0.845 |
| No | 1917 | 255(99.6%) | 1339.3 | 19.0(16.8-21.3) |  |
| Yes | 10 | 1(0.4%) | 6.1 | 16.5(-15.0-48.0) |  |

IRIS, immune reconstitution inflammatory syndrome.
