# Supplementary material for: Machine learning-based in-hospital mortality prediction of HIV/AIDS patients with Talaromyces marneffei infection in Guangxi, China
Source: PLoS Negl Trop Dis. 2022 May 4;16(5):e0010388. doi: 10.1371/journal.pntd.0010388 (PMC9067679; doi:10.1371/journal.pntd.0010388)
Supplement: S3 Table — (DOCX) [file pntd.0010388.s003.docx]

S3 Table. Laboratory measures of 1927 HIV/AIDS patients with T.marneffei infection

| Variables（IQR) | All | Survival（n=1671) | Death (n=256) | *p*-value |
| --- | --- | --- | --- | --- |
| CD3^+^ T-cell count | 296(170,555) | 309(176,564) | 250(126.5,492.5) | <0.001 |
| CD4/CD8 ratio | 0.09(0.05,0.17) | 0.09(0.05,0.16) | 0.11(0.07,0.23) | <0.001 |
| CD4^+^ T-cell count | 21(11,50) | 22(11,51) | 21(11,49.5) | 0.867 |
| CD8^+^ T-cell count | 261(146,482) | 271(154,486) | 215(106.5,435.5) | <0.001 |
| ALT (U/L) | 27(16,50) | 27(16,48) | 28(17,56) | 0.155 |
| LDL (mmol/L) | 1.83(1.24,2.44) | 1.86(1.31,2.46) | 1.46(0.88,2.11) | <0.001 |
| Ca (mmol/L) | 2(1.87,2.13) | 2.01(1.88,2.14) | 1.93(1.8,2.07) | <0.001 |
| TG (mmol/L) | 1.61(1.16,2.28) | 1.59(1.16,2.27) | 1.72(1.18,2.39) | 0.233 |
| HDL (mmol/L) | 0.38(0.15,0.71) | 0.42(0.18,0.74) | 0.15(0.1,0.43) | <0.001 |
| CREA (umol/L) | 73.7(60.1,94) | 72.4(59.8,90) | 88.8(63.5,137) | <0.001 |
| AST (U/L) | 55(28,115) | 52(27,108) | 91(46,195) | <0.001 |
| UREA (mmol/L) | 4.9(3.7,7) | 4.8(3.6,6.5) | 7.5(4.8,12.7) | <0.001 |
| UA (umol/L) | 247(181,347) | 243(181,339) | 289(186,428) | <0.001 |
| LDH (U/L) | 336.3(234.9,540.8) | 323.8(228.2,494.3) | 530.2(309.3,917.4) | <0.001 |
| Glu (mmol/L) | 5.67(4.84,6.79) | 5.72(4.93,6.84) | 5.34(4.13,6.29) | <0.001 |
| CHOL (mmol/L) | 2.85(2.17,3.67) | 2.93(2.26,3.73) | 2.33(1.61,3.13) | <0.001 |
| TBIL (umol/L) | 10.1(6.4,19.1) | 9.6(6.2,17.3) | 17.05(8.7,38.6) | <0.001 |
| AST/ALT ratio | 2.03(1.36,3.17) | 1.96(1.31,2.99) | 3.07(1.74,5.2) | <0.001 |
| BUN/CREA ratio | 0.07(0.05,0.09) | 0.07(0.05,0.08) | 0.08(0.06,0.1) | <0.001 |
| CK (U/L) | 58.9(36.4,114) | 58.3(36,107) | 66.4(38.5,179.3) | 0.008 |
| K (mmol/L) | 3.68(3.33,4.06) | 3.67(3.33,4.02) | 3.83(3.37,4.42) | <0.001 |
| IBIL (umol/L) | 4.5(2.8,7.92) | 4.4(2.8,7.5) | 6.35(3.3,11.8) | <0.001 |
| P (mmol/L) | 1.06(0.88,1.26) | 1.05(0.87,1.24) | 1.17(0.94,1.49) | <0.001 |
| Cl (mmol/L) | 98.6(95,102.4) | 99(95.3,102.6) | 96.7(92.4,101) | <0.001 |
| Na (mmol/L) | 135(130.9,138) | 135.4(131,138) | 133(129,137) | <0.001 |
| Ccr (ml/min) | 62.93(47.73,76.09) | 65.37(49.69,77.74) | 44.2(26.49,61.47) | <0.001 |
| STY (mmol/L) | 277.7(268.8,284.16) | 278.8(269.2,284.4) | 273.4(266.3,281.6) | <0.001 |
| HCO3 (mmol/L) | 24.1(21.3,26.5) | 24.4(21.7,26.5) | 22.6(18.3,25.9) | <0.001 |
| Cys-C (mg/L) | 1.29(1.08,1.65) | 1.25(1.05,1.57) | 1.74(1.33,2.47) | <0.001 |
| AG (mmol/L) | 15.6(12.79,18) | 15.4(12.6,17.7) | 16.65(13.7,21) | <0.001 |
| DBIL (umol/L) | 5(3,12.1) | 4.7(2.9,9.9) | 8.95(4.5,27.1) | <0.001 |
| TBA (umol/L) | 9.1(4.4,23.6) | 8.7(4.3,21.2) | 17(5.4,44.8) | <0.001 |
| WBC (10^9^/L) | 4.3(2.87,6.17) | 4.25(2.87,6) | 4.73(2.78,8.05) | 0.008 |
| RBC (10^12^/L) | 3.33(2.73,3.9) | 3.35(2.76,3.89) | 3.14(2.49,3.9) | 0.029 |
| Hb (g/L) | 91(75,107) | 92(75.4,107.5) | 84.5(66,104.5) | <0.001 |
| PLT (10^9^/L) | 123(59.4,212) | 131(66.4,223) | 64.5(33.7,135.5) | <0.001 |
| MONO (10^9^/L) | 0.23(0.12,0.42) | 0.24(0.13,0.42) | 0.19(0.1,0.39) | 0.006 |
| MONO% (%) | 5.7(2.9,9.6) | 5.9(3.04,9.8) | 4.3(2.1,7.65) | <0.001 |
| RDW-CV (%) | 15(13.6,17.41) | 14.9(13.6,17.21) | 16.11(14.3,18.61) | <0.001 |
| RDW-SD (fL) | 46.2(41.5,53) | 46(41.4,52.8) | 47.3(42,55.71) | 0.023 |
| MCV (fL) | 85.52(79.2,91.1) | 85.7(79.4,91.3) | 83.96(77.22,89.8) | 0.028 |
| HCT | 27.8(22.94,32.67) | 28.1(23.42,32.8) | 25.96(21.05,31.5) | <0.001 |
| LYNPH% (%) | 12.6(7.8,20.1) | 12.92(7.92,20.2) | 11.36(6.37,19.95) | 0.018 |
| LYMPH (10^9^/L) | 0.51(0.3,0.85) | 0.52(0.31,0.84) | 0.45(0.26,0.95) | 0.239 |
| MCH (pg) | 28(25.5,30) | 28(25.6,30.1) | 27.6(24.96,29.6) | 0.016 |
| MCHC (g/L) | 325(313,337) | 325(314,337) | 326(309.6,339) | 0.888 |
| MPV (fL) | 9.6(8.7,10.5) | 9.54(8.6,10.5) | 9.72(8.8,10.7) | 0.142 |
| BASO (10^9^/L) | 0.01(0.01,0.03) | 0.01(0.01,0.03) | 0.02(0,0.04) | 0.029 |
| BASO% (%) | 0.4(0.14,0.7) | 0.4(0.14,0.7) | 0.4(0.14,1) | 0.033 |
| EOS (109/L) | 0.04(0.01,0.11) | 0.05(0.01,0.11) | 0.03(0.01,0.09) | <0.001 |
| EOS% (%) | 1.1(0.34,2.6) | 1.1(0.4,2.8) | 0.84(0.2,1.64) | <0.001 |
| PDW (fL) | 15.5(11.8,16.2) | 15.5(11.7,16.2) | 15.7(14.4,16.5) | <0.001 |
| PCT (%) | 0.13(0.07,0.2) | 0.14(0.08,0.21) | 0.071(0.033,0.15) | <0.001 |
| NEUT% (%) | 78.3(65.1,86.3) | 77.8(64.74,86) | 81.32(68.17,88.45) | 0.010 |
| NEUT (10^9^/L) | 3.14(1.94,4.81) | 3.09(1.94,4.7) | 3.55(1.94,5.94) | 0.024 |
| CRP | 47(14.4,91.7) | 44.1(13.4,87.6) | 73.2(24.2,112.3) | <0.001 |
| hsCRP | 0(0,0) | 0(0,0) | 0(0,0) | 0.002 |
