# Supplementary material for: Machine learning-based in-hospital mortality prediction of HIV/AIDS patients with Talaromyces marneffei infection in Guangxi, China
Source: PLoS Negl Trop Dis. 2022 May 4;16(5):e0010388. doi: 10.1371/journal.pntd.0010388 (PMC9067679; doi:10.1371/journal.pntd.0010388)
Supplement: Attachment — Submitted filename: Response to reviewers.docx [file pntd.0010388.s004.docx]

Dear Roderick Hay and Ahmed Fahal,

Many thanks for the letter dated Jan 11, 2022, regarding our manuscript “***Machine learning-based in-hospital mortality prediction of HIV/AIDS patients with Talaromyces marneffei infection in Guangxi, China*”** (Manuscript # PNTD-D-21-01658). We appreciate the valuable comments of the editor and reviewers. Our point-by-point responses to these comments are as follows.

**Reviewer #1:**

**(1) The objectives of the study were clearly articulated with a clear testable hypothesis stated. The study design was basically appropriate to address the stated objectives. The population was clearly described and appropriate for the hypothesis being tested. No calculation of the sample size for ensuring the adequate power to address the hypothesis being tested. The statistical analysis used to support conclusions were correct in general.**

***Response:*** Thank you for your recognition. We thank the reviewer for pointing out this important issue. You have raised a necessary and important question in the data design process, and we thank you for your question. Our study was implemented based on sample size calculations, and the following is the calculation process we used and added to the text (Page 6-7, Lines 115-123). The sample size was calculated based on the equation as follows:

$n=\frac{(Z_{1-\frac{\alpha}{2}}\sqrt{2‾q‾p}+ Z_{\beta}\sqrt{p_{0}q_{0}+p_{1}q_{1}} )2}{{(p_{1}-p_{0})}^{2}}$ [1]

where Z_α_ represents the standard normal distribution bound, α was set as 0.05, Z_α_ was set as 1.96, and Z_β_=1.282. Generally, the number of exposed groups was designed to be equal to the number of control groups. According to the data previously reported in the literature, the mortality rate of AIDS patients without comorbid *T. marneffei* was p_0_=0.076, and the mortality rate of AIDS patients with comorbid *T. marneffei* was p_1_=0.175[2]. p=1/2（p_0_+p_1_), q_0_=1-p_0_，q_1_=1-p_1_，q=1-p. The sample size was chosen as 233 based on the equation. Our number of cases in the two groups is 256 and 1671, respectively, which can meet the sample requirement. We also collected as many samples as we could base on our ability to meet the minimum sample size requirements to ensure statistical efficacy. In fact, all the samples we could find were included.

*1. Z S, L D, T H. Epidemiology. Beijing: People’s Medical Publishing House; 2017.*

*2.	Jiang J, Meng S, Huang S, Ruan Y, Lu X, Li JZ, et al. Effects of Talaromyces marneffei infection on mortality of HIV/AIDS patients in southern China: a retrospective cohort study. Clinical microbiology and infection: the official publication of the European Society of Clinical Microbiology and Infectious Diseases. 2019;25(2):233-41.*

**(2) Many factors are potentially associated to the prognosis of of AIDS related Talaromycosis, however many important indicators were not included in this article except the lab indexes, such as, the time from onset to diagnosis, the antifungal regimen,** **the time of fungal culture turning positive,** **the types and number of the comorbidities**

***Response:*** We appreciate your comments. The reviewer made very good suggestions, and we quite agree. In fact, the time from onset to diagnosis, the antifungal treatment regimen, and the time of positive fungal culture are more comprehensive information which we unfortunately do not have for various reasons. These are indeed the limitations of our study design, which we also discuss in the Discussion section (Page 23, Lines 418-430). Our main concern is to use some of the most routine test results (blood routine, biochemistry, CD cell level, etc., in this paper) and some of the complications/coinfections data to determine whether there are high-risk mortality factors and to predict patient death by means of machine learning to help clinicians determine whether to classify the patient as a key concern within a relatively short period of time after admission to avoid neglect and delayed treatment. In addition, because the onset of *T. marneffei* is relatively insidious, there are no specific signs and symptoms in the early stage, making the pathogen difficult to identify and the time from onset to diagnosis is difficult to judge. Regarding the current diagnostic tools, the time required for a positive fungal culture is usually 14-20 days after admission to collect a specimen for pathogenic culture, while we largely prognose the patient before then to avoid overlooking the possibility that the patient may have a high risk of death during the waiting process.

**(3) The article listed many lab indicators but ignored the significance and the mutual relationship of these indicators. For example, ALT and AST represent the liver function and would be better to combine for analysis.**

***Response:*** Thank you for your insightful suggestion. As you said, there is an interrelationship between some of the indicators, so we have tried to revert to using the most practical clinical application as much as possible; for example, the AST levels and ALT levels reflect liver function, and a change in a single value may not be indicative, so we have also kept some ratios to synthesize the interrelationship between indicators, such as AST/ALT and CD4/CD8. A decreased AST/ALT ratio points to hepatogenic damage. Conversely, an increase in this ratio is more likely to point to cardiogenic damage. We have revised the statements in accordance with the suggestion in the manuscript: “The elevated ALT level or decreased AST/ALT indicates the possibility of liver damage, and the higher rise in the elevated AST level or AST/ALT may indicate that the myocardium is also involved” (Page 23, Lines 379-386). Thank you for pointing out this omission.

**(4) The observation end point and time, the diagnosis criteria of talaromycosis and other OIs were not explained clearly in this article.**

***Response:*** We thank the reviewer for these comments. The endpoint of our observation is the time of discharge of the patient, and we stopped observation if the patient died during this period (this definition has been added to Page 6, Lines 106-107). Regarding the diagnosis of *T. marneffei*, we wrote in the manuscript (Page 6, Line 109). *T. marneffei* was isolated and cultured from blood, skin tissue, bone marrow, lymph nodes, and/or other bodily fluid samples (mycelia at 25 ℃ and yeast-like structures at 37 ℃); this is by far the most common diagnostic method. For other OIs and complications, we truly did not explain clearly, and that was our mistake. We are very sorry that it is difficult for you to read due to the lack of a complete presentation of our definition. In the revised manuscript, we have added the following diagnostic criteria (Page 7-8, Lines 129-139): Fever was defined as a single oral temperature ≥ 38.3℃ (armpit temperature ≥ 38.0℃) or oral temperature ≥ 38.0℃ (armpit temperature ≥ 37.7℃) lasting more than 1 hour. The diagnosis of pneumonia includes bacterial pneumonia, viral pneumonia, pulmonary mycosis (including Pneumocystis pneumonia) and pneumonia caused by other factors but does not include pulmonary tuberculosis pneumonia, classified as tuberculosis [3]. The definition standard of anemia is as follows: male hemoglobin concentrations of 120 g/L, female hemoglobin concentrations of 110 g/L. Meningitis included purulent meningitis, cryptococcosis meningitis [4], and viral meningitis but did not include tuberculosis meningitis, which is classified as tuberculosis. Coinfections were confirmed according to the diagnostic criteria of chronic hepatitis (hepatitis B or hepatitis C) and oral candida infection found in infectious diseases [5]. The diagnostic criteria of residual complications or coinfections were defined according to the standard of Internal Medicine [6].

*3.	Y M, L Z, Y P. Tuberculosis. Beijing: People’s Medical Publishing House; 2006.*

*4.	J J, S C. Neurology. 7th ed. Beijing: People’s Medical Publishing House; 2013.*

*5.	L L, H R. Infectious diseases. 8th ed. Beijing: People’s Medical Publishing House; 2013.*

*6.	J G, Y X. Internal medicine. 8th ed. Beijing: People’s Medical Publishing House; 2013.*

**(5) The analysis presented match the analysis plan, and the results were clearly and completely presente. however, some figures and tables could be** **merged and simplified to improve the expression efficacy.**

***Response:*** We appreciate your comments. The reviewer made very good suggestions, and we quite agree. We merged Figures 3-6 into Figure 3, which displays the feature engineering results for filtering machine learning predictive model variables, and updated Table S2. The revised manuscript has a total of six figures.

**(6) In general, the conclusions were supported by the data presented. The limitations of analysis were not clearly described here.**

***Response:*** We appreciate your comments. As you mentioned in question (2), we did not collect complete data information. We have added a discussion of these limitations to the revised version of the manuscript: “In fact, the time from onset to diagnosis, the antifungal treatment regimen, the time of fungal culture positivitiy, the types and number of the other comorbidities, the antifungals used and the timing of their administration, any delays in diagnosis after admission, the severity of coinfections, and the timing of antiretroviral therapy, which are more comprehensive information, we unfortunately, for various objective reasons, did not include. We could not control for confounding factors, such as viral load and antifungal therapy, because these data were not collected in the database. Second, our data were only from one hospital, and there were still no external validation data for this hospital, which is the largest HIV/AIDS treatment center in Guangxi Province. The model we built will guide mortality prediction in this hospital. It is a remarkable fact that we did not have data from external validation. Our model was validated internally and maintained a good and stable level of discrimination for the explored outcome. Finally, the data we used are cross-sectional data, but it is noted that the data can be updated in real time when truly applied to the clinic, and further efforts will have to continue to increase the sample size” (Page 24, Lines 428-429).

**(7) The authors have discussed how these data can be helpful to advance our understanding of the topic under study and addressed the relevant public health issue. For some contradictory results for instance“d****eceased patients showed higher levels of CD4/CD8 ratio”, the discussion lacked the reasonable explanation.**
***Response:*** We thank the reviewer for these comments. Thank you for your question. Our description of the limitations is not comprehensive enough, and we lack discussion on the interpretation of some contradictory results. In response to the example mentioned regarding the finding that "deceased patients showed higher levels of CD4/CD8 ratio," from the data of S2, we can clearly see that the median CD4+ T-cell count was 22 in surviving patients and 21 in dead patients (p>0.05), while the median CD8+ T-cell count was 271,215 (p<0.001). This shows that the main reason deceased patients showed higher levels of CD4/CD8 ratio was the lower levels of CD8+ T-cells. We have also added discussion and explanation of this result to the Discussion section (Page 22, Lines 394-399). Thank you again for your suggestion.

**(8) The case numbers in the article were not consistent. For example, table S1 and S2.**

***Response:*** Thank you for your comment. Thank you for pointing out this mistake. We have carefully rechecked and corrected this error in the revised manuscript.

**(9)** The English need to be polished.

***Response:*** Thank you for your suggestion. We had our manuscript linguistically edited and polished by AJE. (This certificate was issued on February 25, 2022 and may be verified on the AJE website using the verification code B7E2-39E7-4CE1-C33F-58D5 .)

**(10) This is an interesting study on in-hospital mortality predictors of AIDS related Talaromycosis by Machine learning. In general, the study has very good originality and the statistical methods are appropriately applied. However, there are quite a few contents that need to be considered and clarified in this article.
*Response:*** We thank the reviewer for these comments. According to some suggestions put forward by the three reviewers, we carefully examined and revised them.

**Reviewer #2:**

**(1) The aim is clearly stated and a large cohort of patients has been used to address the objectives. There is a fairly well described cohort, although there are issues as to when the individual data were obtained in relation to each patient's illness. The study design (use of machine learning) is appropriate although more details of each method of machine learning would be useful. The statistical approach seems reasonable. There are no ethical or regulatory issues.**

***Response:*** We appreciate your comments.

**(2)** **The authors analysed 4 different types of machine learning after having selected the 15 most important variables. There was an emphasis on laboratory variables, possibly because they were easier to obtain from the patient records. This might have introduced some bias away from clinical features, which are often poorly recorded compared to lab data. The results section is slightly difficult to follow as there seemed to be confusion between complications of Talaromyces (e.g. anaemia) and co-infections (e.g. tuberculosis).**

***Response:*** We sincerely thank you for your careful review and useful comments. It is true that we used easily available data for modeling, which is beneficial for popularizing and using the model. According to the easily available data, we obtained the prediction model with the most optimal performance as often as possible. We apologize for the confusion about complications (e.g., anemia) and coinfections (e.g., tuberculosis), and we did not describe them clearly enough. In analyzing the data, we tried to include as many factors as possible that have an impact on mortality, and we classified them into one category, which is called "complication", but it actually included both complications and coinfections. In the revised manuscript, we modified this to “complications/coinfections"

**(3) There was also inclusion of pneumonia as a variable without discussing the causes, one of which could be talaromyces. Failure to recognise that some of the co-infections, rather than Talaromyces, could have been the cause of death in some patients weakens the data set.**

***Response:*** Our aim is to reduce the mortality rate of the population of patients infected with *T. marneffei* who will die from complications, and if we can identify them, we can also reduce the risk of death. Pneumonia could have been the cause of death in some patients. We also included this metric for analysis, but in the feature selection and data preprocessing process, the reasons identified for exclusion according to feature importance resulted in their elimination from the final model. As we replied in the question regarding the fact that “TB and Talaromyces can look similar in HIV, both clinically and radiologically, and pneumonia is a common cause of death in HIV”, the lung lesions of TB are very similar to *T. marneffei* in clinical and radiology, and to mitigate the pneumonia that comes from the combination of TB while treating pneumonia caused by *T. marneffei*, targeted treatment should be taken to reduce deaths.

**(4) Table S2 is a problem: it states that the number of cases is 1956, rather than 1927 stated elsewhere. Also, the number with or without fever (643 and 940 respectively) do not add up to either 1927 or 1956**

***Response:*** Thank you for pointing out the mistakes, and we apologize. We have checked the manuscript and corrected the title of Table S2. For the variable of fever, the total number of cases was not 1927 because some patients were recorded as missing, and we have added the missing cases to Table S2.

**(5) The conclusions appear to be supported by the data, although the machine learning methodology is not clear to me. Some of the limitations are acknowledged but a major flaw seems to be a paucity of clinical data, such as** **what antifungals were used and when,** **any delays in diagnosis after admission, the severity of co-infections, the timing of antiretroviral therapy etc.**

***Response:*** Thank you for your comment. Yes, we did not collect all the useful data, as Reviewer #1 mentioned in the first question. the time from onset to diagnosis, the antifungal regimen, the time of fungal culture positivity and other variables, such as those you mentioned, including the severity of coinfections and the timing of antiretroviral therapy, we would have liked to investigate, but due to objective constraints, we could not obtain this data. This is precisely the limitation of our study, and we also added a discussion of these limitations in the Discussion section (Pages 23-24, Lines 418-430). In the future, we will also focus on the inclusion of these variables, which we believe will be useful for improving the performance of the model.

**(6) There was over-reliance on lab parameters without stating when, in the stage of illness, the blood tests were obtained.** **The authors could be clearer as to how the results can be applied and whether the clinical approach to patients with Talaromyces should be altered as a consequence**

***Response:*** We routinely perform blood tests as soon as a patient is admitted to the hospital before the patient has started formal treatment (Page 6, Line 106). In this way, we obtained the patient's laboratory parameters and then included many clinical features, including comorbidities and coinfections and some representative symptoms (e.g., fever), into our analysis, and we combined these data to predict death in the *T. marneffei* population. This model can be used in prognostic analysis where *T. marneffei* infection is highly suspected or culture positivity reveals *T. marneffei* infection. We have added the following discussion to the revised manuscript: “This brings us to the question of how to reduce deaths in HIV/AIDS patients infected with *T. marneffei*, which is usually accomplished with a change in the method of treatment, including the change of drugs, treatment timing, dosage recommendations, etc., When regarding how these results can be applied, for example, we find that an abnormal AST/ALT level is more likely to cause death, and specifically, a lower ratio points to a high likelihood of liver damage and the necessity of liver protection, while a high ratio necessitates the consideration of heart protection. If a patient with a high likelihood of death is predicted, then the timing of treatment can also be changed, and therapeutic interventions can be made as early as possible” (Pages 23, Lines 404-406).

**(7) Clearly Table S2 needs to be modified. Some of the use of English needs editorial input**

***Response:*** Thank you for your comment. Thank you for pointing out this mistake. We have carefully checked and corrected this error in the revised manuscript.

Thank you for your wonderful suggestion. We had our manuscript linguistically edited and polished by AJE.

**(8)** **Talaromyces is an important complication of HIV infection, particulary in Asia. Mortality rates remain high, often because of late presentation or lack of appropriate therapy. Assessing ways to improve outcomes is, therefore, important both for the individual patient and for public health reasons. Using machine learning to process data to derive prediction models is increasingly being investigated, but the quality of the output depends critically on the data input. This study conflates some variables that are due to the fungal infection with other co-infections that can also be lethal. For example, TB and Talaromyces can look similar in HIV, both clinically and radiologically, and pneumonia is a common cause of death in HIV.**

***Response:*** We appreciate your comments. It is true that patients with *T. marneffei* infections have many similar clinical symptoms to those infected by many other pathogens, which makes early diagnosis of *T. marneffei* difficult, so special clinical attention needs to be paid to the early diagnosis of *T. marneffei*, and the earlier the diagnosis, the more deaths can be reduced. For patients who have been diagnosed with *T. marneffei*, we used various models of machine learning to identify which factors are more predictive of death. This prediction would help clinicians reduce *T. marneffei* patient deaths to some extent. In addition, these confounding variables mentioned here we also discussed in the Discussion section, reminding clinicians to differentially diagnose the symptoms caused by other opportunistic infections, such as TB, which is clinical and radiology similar to *T. marneffei*, and to mitigate the pneumonia that comes from the combination of TB while treating pneumonia caused by *T. marneffei* and take targeted treatment to reduce deaths. (Page 23, Lines 413-417).

**(9)** **If such methods are used to predict mortality, there needs to be some view from the authors as to how such predictions can be used to modify diagnosis and treatment.**

***Response:*** We thank the reviewer for pointing out this important issue. As you mentioned in question: The authors could be clearer as to how the results can be applied and whether the clinical approach to patients with *Talaromyces* should be altered as a consequence, our results can assist physicians in determining the timing and features of early treatment, including the choice of drug, treatment timing, dosage, etc.

**(10)** **It is well known that septic shock and respiratory failure are associated with an increased risk of death, and, as both are obvious clinically, the authors need to explain how the machine learning tool adds value. Clinicians need to be able to identify at-risk patients much earlier, before shock or respiratory failure, to be able to intervene.**

***Response:*** We thank the reviewer for these comments. Septic shock and respiratory failure are associated with an increased risk of death, and our model confirms this. In addition to these two late-stage high-risk clinical symptoms, we also found some predictive laboratory indicators or clinical features, such as platelet and urea levels, which may help identify *the* population with a high risk of death before respiratory failure or shock and thus permits targeted treatment as early as possible, such as by maintaining the normal range of platelet and urea levels. This intervention could reduce the incidence of respiratory failure shock, thereby reducing the incidence of death.

**Reviewer #3:**

**(1) The study need more data to support their hyposis.**

***Response:*** Thank you for your comment. First, as we mentioned, these data come from the hospital electronic medical record information system. We collected as much information as possible, but some data could not be collected. As mentioned by **Reviewer #1,** the time from onset to diagnosis, the antifungal regimen, the time of fungal culture positivity, and the types and number of comorbidities could not be obtained, so this information is missing, which we have also mentioned in the limitations. We have analyzed the data based on that available and obtained some useful results, which are helpful for the treatment of *T. marneffei* patients.

**(2)** **This study attempted to build ML-based prognostic prediction models for HIV/AIDS patients with talaromycosis during hospitalization, the results may have a positive significance for reducing death. However, there are some weaknesses that require further data before acceptance. I have some comments as detailed below:**

**1. The clinical complication and symptom variables described in the study included: “pneumonia, lung infection”, which might be same concepts. As respiratory tract is considered as the initial place for T.marneffei infection, pneumonia or lung infection could be caused by T.marneffei, If so, it should not be considered as clinical complication. This is the first question should be make sense.**

***Response:*** Thank you for your suggestion. We agree with your opinions. In fact, considering exactly this statement, pneumonia, lung infection and pneumocystis have been combined as “pneumonia” in the process of analysis. The description of the manuscript and table was not revised in time following this change. Thank you for pointing out the error we overlooked. We have corrected this error in the revised manuscript.

**(2) 39 laboratory variables were evaluated in the study, It’s unclear when the data was extracted? As the parameters should be changing on the process of the diseas, some good and bad. It should be better assess the outcomes base on the changes of the parameter, rather than certain timepoint’s results.**

***Response:*** Thank you for your careful review and comments. We have indeed considered your suggestion, which is a very good direction, involving dynamic follow-up. At present, we pay attention to the most routinely collected features of the condition of the patient at admission to assess the risk of death because blood and biochemical tests of the patient after admission are common examinations, which constitutes the model data we need here. What we are assessing is the risk of death before the patient receives formal treatment. As a next step, we will try the dynamic follow-up you mentioned.

**(3) The authors mention the AST/ALT ratio in died patients is higher than that in the surviving patients, and suggest elevated ALT level indicate the possibility of liver damage, and the higher rise in the AST level may indicate the myocardium is involved.However, elevated AST and ALT also could be caused by side effect of drugs , especially antifungal agents.**

***Response:*** Thank you for the question. The laboratory data we included are the results of the blood test collected for the first time when the patient was admitted to the hospital (Page 6, Lines 104-106). The patient did not start antifungal therapy and did not exhibit liver damage caused by the drug at that time.

**(4) The sentence in line 330-331, p27, “The number and in-hospital mortality of talaromycosis among HIV admissions increased from 45 and 18.4% in 2012 to 13 and 12.9% in the first half of 2019, respectively”. The mortality increased or decreased within these years? The results seem in contrary with the data.
There are too many spelling and grammatical errors, It requires professional p**

***Response:*** Thank you for pointing out this mistake. We are very sorry that we made a mistake in the use of words and wrote the “decrease” as “increased”. We have corrected this error in the revised manuscript. In fact, according to the data, the overall mortality rate has fluctuated in recent years. The number of deaths and the overall in-hospital mortality rate showed a downward trend (*p*=0.021). Due to the improvement of treatment methods, although it has decreased compared with that of 2012, the lowest rate is still 9.8%. This rate is still very high and worthy of clinical attention, which is also the purpose and reason for us to develop a machine learning model.

Thank you for your letter and for the reviewers’ comments concerning our manuscript. These comments are all valuable and very helpful for revising and improving our paper, as well as the important guiding significance to our research. We have studied the comments carefully and have made corrections that we hope will meet with approval.

Sincerely Yours,

Junjun Jiang, Ph. D

Professors

Guangxi Medical University
