# Supplementary material for: Machine learning-based in-hospital mortality prediction of HIV/AIDS patients with Talaromyces marneffei infection in Guangxi, China
Source: PLoS Negl Trop Dis. 2022 May 4;16(5):e0010388. doi: 10.1371/journal.pntd.0010388 (PMC9067679; doi:10.1371/journal.pntd.0010388)
Supplement: Attachment — Submitted filename: Response to reviewers.docx [file pntd.0010388.s005.docx]

Dear Roderick Hay and Ahmed Fahal,

Many thanks for the letter dated Jan 11, 2022, regarding our manuscript “***Machine learning-based in-hospital mortality prediction of HIV/AIDS patients with Talaromyces marneffei infection in Guangxi, China*”** (Manuscript # PNTD-D-21-01658). We appreciate the valuable comments of the editor and reviewers. Our point-by-point responses to these comments are as follows.

**Reviewer #3**

**(1) The authors mentioned "the elevated AST level may be a manifestation of myocardial damage", which need other biomarkers to support the hypothesis, such as other mycocardial enzymes and PNP. Only base on elevated AST couldn't imply myocardial damage.**

***Response:*** Thank you for your recognition. We appreciate the reviewer pointing out this important issue, and we certainly agree. The discussion of this point in the previous manuscript was not well considered, and in the revised manuscript we made the following modifications: “We rank the contribution of all the independent variables, the AST/ALT ratio was the highest in the feature contribution ranking, we found that patients who died had significantly higher AST/ALT ratios compared with those who survived (3.07 versus 1.96). The previous study has shown an elevated AST/ALT ratio in *talaromycosis* patients.[1]. Two other studies also showed abnormal changes in AST or ALT levels in HIV/AIDS patients with *talaromycosis* [2, 3]. In fact, other fungal studies have also found this phenomenon, a study suggested that the mean ratio of AST to ALT in patients with disseminated histoplasmosis (A fungal disease) was higher than localized pulmonary disease and other endemic mycoses [4]. As we know, ALT is primarily distributed in the liver, kidneys, heart, and skeletal muscle, while AST is primarily distributed in the heart, liver, skeletal muscle, and kidneys. Given our results, AST/ALT ratio may be a predictor of death. Nevertheless, *talaromycosis* is a disseminated disease, the exact site of the damage, the cause of AST and AL changes, and the biological mechanism in *talaromycosis*, which deserves further research.” (Pages 22-23, lines 398-410)

1. *Le T, Wolbers M, Chi NH, Quang VM, Chinh NT, Lan NP, et al. Epidemiology, seasonality, and predictors of outcome of AIDS-associated Penicillium marneffei infection in Ho Chi Minh City, Viet Nam. Clinical infectious diseases : an official publication of the Infectious Diseases Society of America. 2011;52(7):945-52.*
2. *Yousukh A, Jutavijittum P, Pisetpongsa P, Chitapanarux T, Thongsawat S, Senba M, et al. Clinicopathologic study of hepatic Penicillium marneffei in Northern Thailand. Archives of pathology & laboratory medicine. 2004;128(2):191-4.*
3. *Kawila R, Chaiwarith R, Supparatpinyo K. Clinical and laboratory characteristics of penicilliosis marneffei among patients with and without HIV infection in Northern Thailand: a retrospective study. BMC infectious diseases. 2013;13:464.*
4. *Spec A, Barrios CR, Ahmad U, Proia LA. AST to ALT Ratio is elevated in disseminated histoplasmosis as compared to localized pulmonary disease and other endemic mycoses. Medical mycology. 2017;55(5):541-5.*

**(2) The presented data couldn't completly support the conclusions.**

***Response:*** Thank you for your comment. We have modified the conclusion: “The XGBoost ML model is a good predictor in hospitalization outcome of HIV/AIDS patients with T. marneffei infection. The model may have potential application in mortality prediction and high-risk factor identification in the *talaromycosis* population.”

**(3) The revised version is better than the primary version, however it also need further revise.**

***Response:*** Thank you for your comment. We have modified the discussion and conclusion.

Thank you for your letter and for the reviewers’ comments concerning our manuscript. These comments are all valuable and very helpful for revising and improving our paper, as well as the important guiding significance to our research. We have studied the comments carefully and have made corrections that we hope will meet with approval.

Sincerely Yours,

Junjun Jiang, Ph. D

Professors

Guangxi Medical University
